# Dosimetric comparison of helical tomotherapy and volumetric modulated arc therapy in hippocampal avoidance whole‐brain radiotherapy

**DOI:** 10.1002/acm2.14218

**Published:** 2023-11-27

**Authors:** Huai‐wen Zhang, Bo Hu, Hao‐wen Pang

**Affiliations:** ^1^ Department of Radiotherapy Jiangxi Cancer Hospital The Second Affiliated Hospital of Nanchang Medical College NHCKey Laboratory of Personalized Diagnosis and Treatment of Nasopharyngeal Carcinoma Nanchang China; ^2^ Department of Oncology, The third people's hospital of Jingdezhen The third people's hospital of Jingdezhen affiliated to Nanchang Medical College Jingdezhen China; ^3^ Key Laboratory of Nondestructive Testing of Ministry of Education Nanchang HangKong University Nanchang China; ^4^ Department of Oncology The Affiliated Hospital of Southwest Medical University Sichuan China

**Keywords:** dosimetry, helical tomotherapy, hippocampal avoidance, volumetric modulated arc therapy, WBRT

## Abstract

**Objective:**

This study aimed to discuss the dosimetric advantages of helical tomotherapy (HT) and volumetric modulated arc therapy (VMAT) technology in hippocampal avoidance whole‐brain radiotherapy and provide references for clinical selection of ideal radiotherapy technology.

**Methods:**

A total of 20 patients with hippocampal avoidance whole‐brain radiotherapy were chosen randomly. Computed tomography (CT) and MRI scanning images were input into the treatment planning system (TPS). After the CT and enhanced magnetic resonance T1 weighted images were fused and registered, the same radiation therapy physician was invited to outline the tumor target volume. PTV‐HS refers to the whole brain subtracted by 5 mm outward expansion of the hippocampus (HP). The prescribed dose was 30 Gy/10 fractions. HT and VMAT plans were designed for each patient in accordance with PTV. Under the premise that the 95% isodose curve covers the PTV, dose–volume histogram was applied to evaluate the PTV, conformal index (CI), heterogeneity index (HI), maximum dose (Dmax), mean dose (Dmean), minimum dose (Dmin) and absorbed doses of organs at risk (OARs) in HT and VMAT plans. Paired *t*‐test was performed to compare the differences between two radiation therapy plans, and *p*  <  0.05 was considered statistically significant.

**Results:**

These two plans had no significant difference in PTV‐HS (max, min, and mean). However, the HI and CI of the HT plan were significantly better than those of the VMAT plan, showing statistically significant difference (*p* < 0.05). The HT plan was significantly superior to the VMAT plan in terms of the Dmax, Dmin, and Dmean of HP, left and right eye lens, left and right eye, and spinal cord, showing statistically significant difference (*p* < 0.05). The HT plan was also better than the VMAT plan in terms of the Dmax of the left optic nerve. However, the two plans showed no obvious differences in terms of the absorbed doses of the right optic nerve and brainstem, without statistical significance.

**Conclusions:**

Compared with the VMAT plan of hippocampal avoidance, HT technology has significant dosimetric advantages. HT plans significantly decreased the radiation dose and radiation volume of OARs surrounding the target area (e.g., surrounding eye lens and eye, especially hippocampal avoidance area) while increasing the CI and HI of PTV dose in whole brain radiotherapy (WBRT) greatly, thus enabling the decrease in the incidence rate of radioactive nerve function impairment.

## 1 INTRODUCTION

The progress risks of intracranial metastasis in patients without whole brain radiotherapy (WBRT) are 70%−300% higher than those in patients with WBRT.^1^, ^2^ The European Organization for Research and Treatment of Cancer has proven through a test (# 22952−26001)that the local failure rates with and without WBRT are 23% and 42%, respectively.^3^ With the comprehensive development of radiotherapy, prophylactic cranial irradiation (PCI) has become a standard therapeutic scheme for primary intracranial tumor and intracranial metastatic tumor. It could not only relieve symptoms effectively but also significantly increase and improve the survival effect of patients. Slotman et al. proved that PCI could decrease the brain metastasis rate in patients with non‐small cell lung cancer (NSCLC) from 40.4% to 14.6% and increase the 1‐year overall survival (OS) from 13.3% to 27.1%.^4^ However, WBRT often causes impairments to neurocognitive functions and cerebellar functions of patients.^5^ The RTOG 0214 phase III pointed out that although PCI could control intracranial metastatic tumor effectively, it significantly decreases short‐term memory and delayed memory of patients, because the tolerant dose of parahippocampal gyrus is low, and radiation causes neural stem cell injuries in the subgranular region of hippocampus (HP) granules, thus resulting in obvious hypomnesis. Gondi et al.^6^ found that patients showed deteriorated memory function after > 7.3 Gy irradiation to 40% volume of bilateral HP. Bernad et al.^7^ analyzed the volume changes in the parahippocampal gyrus and amygdaloid body of patients with small cell lung cancer (SCLC) before and after standard treatment through brain MRI. They revealed that chemo‐radiotherapy could cause structural damages to the parahippocampal gyrus and amygdaloid body.

With the prolonging of patients’ OS, the neurocognitive impairment caused by WBRT is receiving more and more attention.^8^ A study has pointed out that WBRT could control intracranial tumor well, but radiotherapy could damage hippocampal neural stem cells, thus influencing hippocampal functions and brain tissue repair, which is the major cause of cognitive disorders, such as memory loss.^9^ RTOG 0933 carried out a multicenter phase II clinical test to compare WBRT with and without hippocampal avoidance. The results showed that the Hopkins verbal learning test (HVLT) delayed recall of WBRT with hippocampal avoidance was 7%, which was far lower than that in WBRT without hippocampal avoidance.^10^ WBRT with hippocampal avoidance is better than the standard WBRT in maintaining neurocognitive functions and improving the quality of life of patients.^11^ It has become a method to maintain control over intracranial tumor and minimize cognitive dysfunction.^12^, ^13^, ^14^

In order to ensure the conformal index (CI) and heterogeneity index (HI) in the planning target volume (PTV) under WBRT, while significantly reducing the irradiation dose and irradiation volume of the HP avoidance area, many researchers have used methods such as dividing the tumor target volume into multiple segments, using three or more radiation fields, and using tilted headrests or non coplanar radiation fields for radiotherapy plan design.^15^, ^16^, ^17^ Although intensity modulated radiotherapy (IMRT) and volumetric modulated arc therapy (VMAT) could protect HP to some extent, some studies have proven that they could not meet the HP maximum dose (Dmax) < 17 Gy and D100% < 10 Gy in the RTOG 0933 report. Helical tomotherapy (HT) therapy has great advantages of small segments, multiple angles, and strong intensity‐modulated capability. In the present research, the new tomotherapy Accuray's Radixact system was used. VMAT and HT were used to design radiation therapy plans for 20 patients in hippocampal avoidance whole‐brain radiotherapy. The dose distribution, CI, and HI of the target region and the irradiation dose in the organs at risks (OARs), such as HP and eye lens, of these two plans were dosimetrically compared.

## 2 MATERIALS AND METHODS

### 2.1 Patient data

A total of 20 patients who had hippocampal avoidance whole‐brain radiotherapy were admitted in the Department of Radiotherapy from January 2020 to December 2022. The median age was 62.2 (46−79) years. The inclusion criteria were as follows: age > 18 years; recursive partitioning analysis score: level I or II; karnofsky performance score > 70; no obvious neural and mental symptoms; cranial MRI scanning was evaluated in 1 month; no obvious anomalies in blood routine examination, biochemistry, and coagulation indicators; and estimated survival time ≥ 3 months. The exclusion criteria were as follows: metastatic tumor in HP and 5 mm expanded range; history of brain surgery or WBRT treatment; history of mental and cognitive disorders; anomalies in Mini‐Mental State Examination baseline scores (< 27 scores); patients diagnosed or suspicious of meningeal metastases; and patients who could not complete the contrast enhancement scanning due to moderate and severe abnormal renal function.

### 2.2 Posture fixation and computed tomography (CT) scanning

A piece of thermoplastic facial mask was fixed at the head of the patients. CT scan orientation under free calm breathing conditions was performed under the SOMATOM Definition AS 20 spiral CT. The direction perpendicular to the long axis of the patients’ bodies was chosen as the scanning baseline, and scanning slice thickness was set at1.25 mm. In the scanning, field of view (FOV), voltage, and current were set as 25 cm, 120 kV, and 540 mAs, respectively. The scanning range was from calvarium to the third cervical vertebra (C3). MRI scanning at the same position was performed in accordance with the CT simulated (Philips Ingenia 3.0T MR‐sim) without a position fixing mask. The scanning range was from the base of the skull to the top of ventricle. The 3D spoiled gradient echo, standard axial position and coronal FLAIR sequence, and axial T2 weighted and gadolinium contrast‐enhanced T1 weighted sequences were obtained. The slice thickness was also set at 1.25 mm. The CT and MRI images were transmitted to the Eclipse (Varian Medical Systems, Palo Alto, CA) 3D radiotherapy planning system through the radiotherapy special local area network.

### 2.3 PTV and OARs

Three‐dimensional reconstruction was implemented on the patients’ CT images, which were then input into Eclipse, followed by Downhill Simplex fusion and registration algorithm with contrast enhanced magnetic resonance T1 weighted images. HP was outlined by the same radiotherapist on the fused images in accordance with the RTOG0933 report. The parahippocampal gyrus region is a low‐signal gray structure. It is generally crescentiform starting from the crescent base of lateral ventricles, with the leading edge of temporal angle as the anterior boundary of the HP and the lateral ventricular cerebrospinal fluid as the external boundary. The outlined HP was extended by 5 mm in the 3D directions to form HP protection‐PRV (HP‐PRV) to realize the rapid decrease in dose from PTV to HP. PTV is generally outlined as the whole brain, mainly including bilateral brains, cerebella, and brainstems, with the lower boundary to the odontoid layer as the clinical target volume. PTV‐HS refers to the whole brain subtracted by 5 mm outward expansion of the HP, and it was the actual planned target volume. The OARs mainly include eye lens, eye, optic nerves, and brainstem.

### 2.4 Design and implementation of plans

The treatment plan based on the overall PTV was designed by medical physics experts on the physicist workstation of the Eclipse (version 13.5, Varian Medical Systems, Palo Alto, CA) and the HT (version precise1.0, Accuray Inc., Madison, WI) treatment planning system (TPS) after confirmation by radiotherapist. VMAT and HT plans were designed for each patient. VMAT plans generated using 6 MV flattening filter photons beams of the Varian Truebeam linear accelerator (Varian Medical Systems, Palo Alto, CA), and the max dose rate was set at 600 MU/min. HT plans were generated using 6 MV flattening filter‐free photons beams of Accuray Radixact (Accuray Inc., Madison, WI), and the dose rate was set at 850 MU/min. The patients were treated with postoperative radiotherapy to a prescribed dose of 30 Gy in 3 Gy fractions 5 days per week. The two plans were prescribed such that 95% of the whole brain PTV received the prescription dose of 30 Gy. In order to achieve objectivity in dosimetric comparisons, we normalize 95% of the target volume to 30 Gy. While there are differences in the dose calculation algorithms for each of the two type planning systems used in this study, such difference is minimal relative to the dosimetric differences from the delivery modalities investigated in this study. The PTV volume receiving more than 110% of the prescribed dose is less than 5%. Meanwhile, the dose limits of OARs were determined in the two plans as follows: Dmax ≤ 37.5 Gy for optic nerve; Dmax ≤ 33 Gy for eye, Dmax ≤ 8 Gy for eye lens; and Dmax < 33 Gy for brainstems; the dose limits of HP may be decreased to the minimum dose D_100%_ < 10 Gy and the Dmax < 17 Gy as much as possible.

1. VMAT plan: The center of field was set at the midline of the bilateral HP, and the max dose rate of the radiation field was set at 600 MU/min. Two full‐arc coplanar irradiations were applied. The rotation angle of one arc was rotated from 179° to 181° along counter‐clockwise direction. The rotation angle of the other arc was rotated from 181° to 179° along clockwise rotation. The angles of the two arc collimators were 315° and 45°, and the angle of the treatment table was 0°. The sliding windows jaw follow mode was adopted. Constraints, such as the reverse optimization goal of the total PTV and doses of OARs, were set in the TPS. Photo optimization algorithm was used for optimization, and the final doses were calculated using the Anisotropic Analytical Algorithm . The grid size for dose calculation was 2.5 mm.
2. HT plan: To maximize sparing of the hippocampi while preserving target coverage, the Jaw mode was chosen as dynamic jaw. The field width was 2.5 cm, the pitch was selected as 0.185 cm, and the beam intensity modulated factor (MF) was set at 3.5. The 360° rotating irradiation mode was applied. The collapsed cone convolution superposition algorithm was used during the full dose and final dose steps. The grid size for final dose calculation was high resolution mode.

### 2.5 Acquisition of dose parameters

The PTV and doses of OARs in the two intensity‐modulated plans were assessed using 3D Dose‐volume histogram (DVH) and relevant dosimetric data provided by TPS.

### 2.6 Observation indices and plan evaluation

The two treatment plans were compared in terms of the HI and CI of the target region and the volume dose of related normal tissues by DVH. The evaluation parameters of PTV‐HS included the following: (1) The mean dose (Dmean), Dmax, and minimum dose (Dmin) of PTV‐HS; (2) HI is the ratio of maximum dose and minimum dose, and it reflects the heterogeneity of dose in PTV. The calculation formula is.^18^ $\mathrm{HI}=D_{5\%}/{D_{95}}_{\%}$, where $D_{5\%}$ and $D_{95\%}$ are the minimum doses at 5% and 95% PTVs, respectively. Higher values indicate poorer heterogeneity of dose in PTV. (3) CI is used to evaluate the conformal degree of PTV and the reference isodose surface. The calculation formula of CI is.^19^ $CI=\left(V_{t,ref}/V_{t}\right)\times \left(V_{t,ref}/V_{ref}\right)$, where $V_{t,ref}$ is the PTV covered by 95% prescribed dose, $V_{t}$ is the total PTV, and $V_{ref}$ is all volumes covered by 95% doses. The CI ranges between 0 and 1. A high CI indicates a good conformity degree. The evaluation parameters of OARs included the Dmax, Dmin, and Dmean of brainstem, eye, eye lens, optic nerves, spinal cord, and HP.

### 2.7 Statistical method

All DVH data were input into and analyzed by SPSS 13.0. Quantitative data were expressed as the mean ± standard deviation ($\bar{x}\pm s$). Paired *t*‐test was performed to compare the differences, with *p* < 0.05 representing statistically significant difference.

## 3 RESULTS

### 3.1 Comparison of PTV‐HS, HI, and CI

The PTV‐HS_(max, min, mean)_, HI, and CI of the target region in two treatment plans are listed in Table 1. Under the premise that the 95% isodose curve covers the PTV‐HS, the two plans had no differences in the Dmax, Dmin, and Dmean of PTV. However, the HT plan was significantly superior to the VMAT plan in terms of HI and CI, showing statistically significant differences (*p* < 0.05).

**TABLE 1:** Comparison of planning target volume Dose, conformal index, and heterogeneity index of two plans ($\bar{x}\pm s$).

Project indicators	HT	VMAT	*t* value	*p* value
PTV‐HS max(Gy)	33.22 ± 0.47	33.43 ± 0.48	−1.432	0.168
PTV‐HS min(Gy)	15.17 ± 3.20	15.43 ± 4.27	−0.222	0.827
PTV‐HS mean(Gy)	31.04 ± 0.38	31.20 ± 0.30	−1.532	0.142
HI	1.05 ± 0.02	1.10 ± 0.04	−6.707	0.000*
CI	0.88 ± 0.02	0.84 ± 0.03	4.835	0.000*

*Note*: ^*^ *p* < 0.05 represents statistically significant difference between the two methods.

### 3.2 Comparison of the irradiation dose and volumes of OARs under the two plans

The absorbed dose parameters of OARs in the two plans are listed in Table 2. The HT plan was significantly superior to the VMAT plan in terms of the Dmax, Dmin, and Dmean in HP, left and right eye lens, left and right eye, and spinal cord, with statistically significant differences (*p* < 0.05). The HT plan was also better than the VMAT plan in terms of the Dmax in the left optic nerves. However, the absorbed dose of the right optic nerve and brainstem of the two treatments plans had no significant difference (*p* > 0.05).

**TABLE 2:** Irradiation doses and volumes in organs at risk of two plans (%, $\bar{x}\pm s$).

Organ	Project indicators	HT	VMAT	*t* value	*p* value
Left Eye lens	D_max_(Gy)	4.19 ± 0.63	7.13 ± 1.35	−11.403	0.000*
	D_min_(Gy)	2.62 ± 0.28	4.98 ± 1.29	−8.590	0.000*
	D_mean_(Gy)	3.11 ± 0.34	5.91 ± 1.20	−11.929	0.000*
Right Eye lens	D_max_(Gy)	4.12 ± 0.46	7.09 ± 1.17	−13.101	0.000*
	D_min_(Gy)	2.61 ± 0.30	5.01 ± 1.02	−12.537	0.000*
	D_mean_(Gy)	3.07 ± 0.37	5.79 ± 1.14	−12.738	0.000*
Left eye	D_max_(Gy)	27.06 ± 2.86	25.58 ± 3.67	3.109	0.006*
	D_min_(Gy)	2.56 ± 0.28	4.73 ± 1.22	−8.096	0.000*
	D_mean_(Gy)	9.89 ± 1.26	11.20 ± 1.73	−4.026	0.001*
Right eye	D_max_(Gy)	27.26 ± 2.91	26.55 ± 3.11	1.903	0.073
	D_min_(Gy)	2.56 ± 0.30	4.72 ± 1.07	−10.735	0.000*
	D_mean_(Gy)	10.02 ± 1.48	12.04 ± 1.64	−5.523	0.000*
Left optic nerve	D_max_(Gy)	29.78 ± 1.07	30.63 ± 1.06	−2.398	0.028*
	D_min_(Gy)	18.14 ± 3.59	18.77 ± 3.13	−0.964	0.348
	D_mean_(Gy)	26.09 ± 1.81	26.35 ± 2.39	−0.584	0.567
Right optic nerve	D_max_(Gy)	29.74 ± 1.33	30.78 ± 1.16	−2.494	0.023*
	D_min_(Gy)	18.89 ± 3.85	19.74 ± 2.95	−1.234	0.235
	D_mean_(Gy)	26.25 ± 1.83	26.59 ± 2.19	−0.797	0.437
Spinal cord	D_max_(Gy)	24.59 ± 8.44	23.81 ± 9.31	1.285	0.216
	D_min_(Gy)	0.27 ± 0.19	0.11 ± 0.09	6.446	0.000*
	D_mean_(Gy)	3.07 ± 1.76	2.40 ± 1.44	3.415	0.003*
Hippocampus	D_max_(Gy)	15.42 ± 1.16	17.13 ± 1.33	−4.781	0.000*
	D_min_(Gy)	7.28 ± 0.76	9.14 ± 0.83	−7.985	0.000*
	D_mean_(Gy)	9.23 ± 0.94	11.77 ± 1.02	−11.323	0.000*
Brainstem	D_max_(Gy)	32.80 ± 0.42	33.08 ± 0.61	−1.648	0.118
	D_min_(Gy)	15.79 ± 5.86	17.33 ± 3.58	−1.344	0.196
	D_mean_(Gy)	29.34 ± 3.09	29.94 ± 0.66	−0.805	0.432

*Note*: ^*^ *p* < 0.05 represents statistically significant difference between the two methods.

## 4 DISCUSSIONS

In this study, two intensity‐modulated plans of HT and VMAT were designed. The differences in the dose distribution of PTV‐HS and OARs in patients receiving WBRT with hippocampal avoidance were analyzed. According to research results, the HT and VMAT plans had no difference in the Dmax, Dmin, and Dmean of PTV‐HS. The PTV‐HS coverage of both plans could meet the practical clinical requirements. However, the HT plan had significant advantages in the CI and HI of PTV‐HS, similar with the research results of Ozdemir Y and X Dong et al.^20^, ^21^ This finding is mainly due to the CI and HI of PTV‐HS being positively related with the plan irradiation fields and number of segments. The HT plan has advantages of thousands of small segments, multiple angles, and strong intensity‐modulated capacity. Each helical period has 51 arc irradiation fields, each arc irradiation field has 64 adjustable multi‐leaf‐collimators, and each subfield has 100 levels of intensity for adjustment.

In the study, the mean HP volume of patients was about 4.1 cm^3^, which was basically consistent with the associated studies in China and other countries.^22^ None of the existing guidelines and literature has enlisted HP as the limit dose of OAR. Hence, the tolerance dose of HP remains very unclear. Many studies.^23^, ^24^have proven that the brain metastasis rate and recurrence rate in HP and surrounding 5 mm were relatively lower, and most cases of brain metastasis occurred on positions far away from the parahippocampal gyrus. Ghia et al.^25^ revealed that removing HP and the surrounding 5 mm region without metastatic tumor in WBRT is reasonable. Hence, the recommendations of the RTOG 0933 report were applied in the present study, that is, the HP + 5 mm region was set as the HP protection area.^26^

Van Kesteren et al.^27^ designed a treatment plan for 10 patients with whole‐brain hippocampal avoidance by using the left‐right pair throughfield. This plan could decrease the Dmean in the hippocampal avoidance area to 6.1 ± 0.4 Gy and decrease the Dmax of HP to 13.5 ± 1.9 Gy. However, the coverage of whole brain target decreased significantly to 81% ± 0.9%. Jiang et al.^28^ compared IMRT plans with and without HP protection among 10 patients with brain metastases of lung cancer. They found that although intensity‐modulated radiotherapy could lower the radiation volume in bilateral HP regions, the maximum dose point of HP decreased slightly. Rong et al.^29^ found that HT demonstrates a better capacity for selective sparing of tissue compared to IMRT. Yuen^30^ and Sood et al. ^31^ proved that VMAT could decrease the dose of HP. Fu et al.^32^ designed a inclined head four full‐arc VMAT plan for 16 patients who had whole‐brain hippocampal avoidance, and the plan could decrease the Dmax of HP. Sun et al.^33^ studied the whole‐brain prophylactic irradiation of 15 cases of NSCLC and found that VMAT could improve HP protection. Zhou et al.^34^ found that HT plan was superior to IMRT plan in craniospinal irradiation with hippocampal avoidance in pediatric patients, but HT had relatively high secondary carcinogenic risks to thyroid and lung. All of these findings reflected that the irradiation dose and volume in the hippocampal avoidance area in the HT plan were significantly lower, without any increase in low‐dose areas. For the patients of the HT and VMAT plans in our study, the doses of HP were as follows: the Dmax was 15.42 ± 1.16 Gy versus 17.13 ± 1.33 Gy, the Dmean was 9.23 ± 0.94 Gy versus 11.77 ± 1.02 Gy, and the Dmin was 7.28 ± 0.76 versus 9.14 ± 0.83. This study's results were correlation with the findings of the aforementioned researches, and the dose of the hippocampus is relatively lower. This is mainly because that the study focused on patients who underwent WBRT rather than the whole brain and central nervous system, and the HT plan had significantly more subfields than the VMAT plan, thus resulting in the faster decrease in dose. The HT plan was also better in terms of the doses of the left and right eye lens and the left and right eye. Our finding agrees with the results of Wang^35^ and Jiang et al.^36^ who compared the hippocampal avoidance effects of WBRT and PCI between HT plan and VMAT plan. However, the HT plan used small pitch and great MF in plan design, and the design time and overall implementation time were longer than those of the VMAT plan, which may bring some influences to the treatment of patients.^37^

## 5 CONCLUSIONS

The HT plan has significant dosimetric advantages in hippocampal avoidance compared with the VMAT plan. It could increase the CI and HI of PTV‐HS dose distribution in WBRT and decrease the irradiation doses and irradiation volumes in OARs, such as surrounding eye lens and eye of PTV‐HS, especially the hippocampal avoidance area, thus decreasing the incidence rate of radioactive neural functional impairment.

## AUTHOR CONTRIBUTIONS

Huai‐wen Zhang conceived of the presented idea. Bo Hu and Hao‐wen Pang collected the planning data of all patients in this study. Huai‐wen Zhang and Hao‐wen Pang took the lead in writing the manuscript. All authors provided critical feed‐back and helped shape the research, analysis, and manuscript.

## CONFLICT OF INTEREST STATEMENT

There are no conflicts of interest to declare.

## ETHICS DECLARATIONS

Ethics approval and consent to participate.According to the ethical guide‐lines of the Helsinki Declaration and was approved by the institutional review board of Jiang‐xi Cancer Hospital(ethics number: 2022KY042). Written informed consents were obtained from all patients prior to treatment.

## CONSENT FOR PUBLICATION

Consent for publication is not applicable in this study, because there is not any individual person's data.

## Data Availability

All data generated and analyzed during this study are included in this published article.
